# Thyroid disorders and COVID-19: a comprehensive review of literature

**DOI:** 10.3389/fendo.2025.1535169

**Published:** 2025-05-19

**Authors:** Narges Anbardar, Shanai Lashayla Dixon, Samhitha Munugoti, Maneesh Gaddam, Kebria Kashfi, Lillian Kasulis, Andrew L. Messersmith, Kamyar Asadipooya

**Affiliations:** ^1^ Department of Clinical Medicine, St Matthew’s University School of Medicine, Grand Cayman, FL, United States; ^2^ Division of Endocrinology, Diabetes, and Metabolism, University of Kentucky, Lexington, KY, United States; ^3^ Pulmonary, Critical Care and Sleep Medicine, Appalachian Regional Healthcare, Hazard, KY, United States; ^4^ Department of Clinical Medicine, American University of Antigua College of Medicine, Coolidge, AG, United States; ^5^ Department of Medicine, Division of Endocrinology, Diabetes, and Metabolism, Barnstable Brown Diabetes and Obesity Center, University of Kentucky, Lexington, KY, United States

**Keywords:** ACE2, hyperthyroidism, hypothyroidism, SARS-CoV-2, thyroiditis

## Abstract

**Background:**

The literature is rapidly evolving with regards to the endocrine consequences of coronavirus disease 2019 (COVID-19), including diabetes, thyroid dysfunction, adrenal and pituitary disorders. There is evidence suggesting that severe acute respiratory syndrome coronavirus 2 (SARS-CoV-2) infection can lead to thyroid dysfunction and long-term sequelae. We aimed to review the current evidence and propose a preventive approach based on the published data since the beginning of the COVID-19 pandemic.

**Methods:**

A comprehensive review of literature was conducted using electronic databases PubMed and Google Scholar. Two authors independently used the keywords “Thyroid, Hypothyroidism, Hyperthyroidism, Graves, Thyroid Eye Disease, or Thyroiditis” and “Coronavirus, SARS-CoV-2 or COVID-19” to search these databases. We screened titles and abstracts for initial selection and then reviewed the full text of relevant studies to report the outcomes of published data.

**Results:**

We selected 28 manuscripts. SARS-CoV-2 infection appears similar to other viruses. It affects thyroid function resulting in non-thyroidal illness syndrome, which usually resolves spontaneously. COVID-19 also causes subacute thyroiditis. It may also trigger autoimmunity against the thyroid that leads to autoimmune thyroiditis. Autoimmune thyroiditis or subacute thyroiditis may progress to clinical or subclinical hypothyroidism and clinical or subclinical hyperthyroidism. Patients with pre-existing thyroid dysfunction probably have similar risks of SARS-CoV-2 related adverse outcomes.

**Conclusions:**

Evaluation of thyroid function is important in COVID-19 patients. Improving the efficacy of treatment against acute SARS-CoV-2 infection can reduce the risks of short-term and long-term complications.

**Systematic Review Registration:**

https://www.crd.york.ac.uk/prospero, identifier CRD42023447994.

## Introduction

The severe acute respiratory syndrome coronavirus 2 (SARS-CoV-2) has profoundly impacted society since the beginning of the coronavirus disease 19 (COVID-19) pandemic. Generally, the SARS-CoV-2 infection can present from mild to severe disease and death. It continues to have adverse effects including damage to the endocrine systems. The route of entry of SARS-CoV-2 is mainly via angiotensin-converting enzyme 2 (ACE2). ACE2 is not only a receptor for SARS-CoV-2, but also has protective roles against organ damage by converting angiotensin II into angiotensin 1–7 and down regulating the renin-angiotensin system (1, 2). However, the increased expression of ACE2 and its cofactors, the host proteases transmembrane protease, serine 2 (TMPRSS2) and A Disintegrin and Metalloproteinase 17 (ADAM17), in human tissues can facilitate SARS-CoV-2 entry into human cells, and therefore, increase the risk of organ damage following SARS-CoV-2 infection, including the thyroid gland (1, 3).

The first case of COVID-19 with thyroid dysfunction was reported in May 2020 by Brancatella et al., which was a case of subacute thyroiditis (SAT) following mild, symptomatic SARS-CoV-2 infection (4). There is also an association between COVID-19 and autoimmune diseases, which raises concern regarding a causal relationship between SARS-CoV-2 infection and autoimmune thyroid diseases (5). Basically, the expression of ACE2 and TMPRSS2 on the thyroid follicular cells enables SARS-CoV-2 to invade thyroid cells, and increases the susceptibility of the thyroid gland to SARS-CoV-2 related injuries (6). Furthermore, long COVID is a debilitating complication of acute SARS-CoV-2 infection, possibly due to persistent SARS-CoV-2 reservoir or immune activation (7). Therefore, long COVID can also be associated with further organ damage, such as endocrine organs including the thyroid gland. Although there are studies reporting almost complete recovery of thyroid function following acute SARS-CoV-2 infection, the overlapping symptoms between long COVID and thyroid dysfunction highlight the point that subtle organ damage should be investigated more carefully with pertinent clinical presentations (8).

Preventive and therapeutic strategies may improve the clinical outcomes of acute SARS-CoV-2 infection. However, differences in race and ethnicity, in addition to social and political factors such as economic inequalities, accessibility to healthcare resources, and vaccine hesitancy, were potential drivers of outcome inequalities in different regions of the world (9, 10). There is a concern exists for SARS-CoV-2 mutations, with resistance to antiviral therapy (11). Therefore, proposing a therapeutic approach that is effective against new strains of SARS-CoV-2, and that can be implemented in developing countries is important to prevent acute and chronic complications of COVID-19 infection.

This review summarizes the incidence of thyroid dysfunction following acute SARS-CoV-2 infection, including outcomes in different countries. Gender and vaccine status are other variables that are considered in this review, and may play a role in susceptibility to thyroid disorders. Finally, this article will discuss the pros and cons of current therapeutic approaches, and introduce a new strategy to mitigate SARS-CoV-2-related complications.

## Materials and methods

### Searches strategies

We conducted a comprehensive review of literature according to PRISMA guidelines, identifying studies via PubMed and Google Scholar (12). The review was registered on the International Prospective Register of Systematic Reviews (PROSPERO; CRD42023447994) (13). The search was limited to English literature. The timeline for searching the database was between January 2020 and March 2024. A comprehensive search was conducted by using standardized terms, such as “Thyroid, Hypothyroidism, Thyrotoxicosis, Hyperthyroidism, Graves’ Disease, Thyroid Eye Disease, Graves’ Ophthalmopathy, Thyroiditis” and “Coronavirus, SARS-CoV-2 or COVID-19”.

### Study selection and data extraction

**Figure 1:**
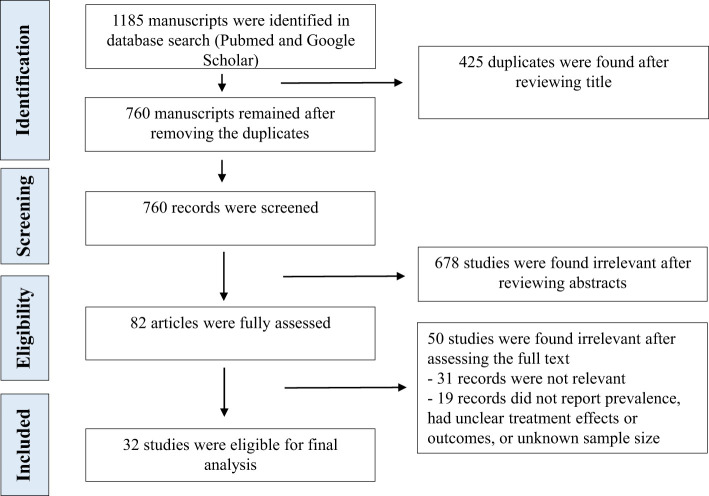
The screening process of reviewing the literature (PRISMA flow diagram).

Two investigators independently searched PubMed and Google by reviewing titles and abstracts. We looked at the studies’ participants, type, outcomes and interventions to select appropriate manuscripts. The full text of selected articles was assessed afterward. The third author made a cross-check to confirm the consistency of search results and exclusion of duplicates or irrelevant studies. We reviewed the published systemic reviews from early 2022 (Deng L 2024, Ganie MA 2024, Lui DTW 2024, Ashrafi S 2024, Lampropoulou E 2024, Singhal V 2023, Wei J 2023, Vamshidhar IS 2023, Lee ZC 2023, Chen K 2023, Takedani K 2023, Meftah E 2023, Bellamkonda A 2023, Li Z 2023, Darvishi M 2022, Triantafyllidis KK 2022, Patrizio A 2022, Ando Y 2022, Tutal E 2022) and references of selected studies to include all potentially relevant manuscripts during the selection process. We looked at [a] clinical research articles, such as clinical trials, cohort, cross-sectional, case-control studies and case series, [b] review articles including mini-reviews, systematic reviews and meta-analyses, [c] opinion and commentary articles, like editorials, commentaries, perspectives, and letters to editor that discussed the incidence and outcomes of thyroid disorders following COVID-19 infection or vaccination. Studies were excluded if they did not provide enough information about the prevalence and outcomes of thyroid disorders following acute SARS-CoV-2 infection. We finally reported a summary of the results of the clinical research articles (**Tables 1**, **2**), such as retrospective, cross-sectional, and prospective studies, which include the first author, journal, year of publication, country of origin, study design, number of participants, type of intervention, age, gender, outcomes, type of thyroid disorders and other relevant results. We considered sex and vaccine status to assess high risk-populations for SARS-CoV-2 infection induced thyroid disorders. We excluded case reports and case series with less than 10 cases. **Figure 1** shows the flowchart for our review.

**Table 1:** Observed thyroid dysfunction following acute SARS-CoV-2 infection.

Country	Thyroid Autoimmunity	Non-thyroidal Illness	Thyroiditis	Subclinical Hypothyroidism	Hypothyroidism	Subclinical Hyperthyroidism	Hyperthyroidism or Graves’ disease
Bangladesh (14)		Yes					
Bosnia and Herzegovina (15)				Yes	Yes		
Bulgaria (16)	Yes			Yes	Yes	Yes	
Castilla y León, Spain (17)							Yes
China (18–22)	Yes	Yes	Yes	Yes		Yes	Yes
Columbia (23)	Yes						
Hong Kong (24, 25)	Yes	Yes	Yes			Yes	
Hungary (26)	Yes		Yes				
India (27, 28)		Yes	Yes	Yes	Yes		
Italy (29–31)	Yes		Yes	Yes	Yes	Yes	Yes
Italy (32)			No				
Nepal (33)				Yes			
Pakistan (34)							
Qatar (35)	Yes	Yes	Yes		Yes		Yes
Romania (36)							
Saudi Arabia (37)		Yes					
South Korea (38)			Yes				
Turkey (39)							
United Kingdom (40–42)		Yes					
USA (43)							Yes

The countries where studies were conducted are listed in alphabetical order.

**Table 2:** Summary of the outcomes of research about thyroid dysfunction following acute SARS-CoV-2 infection, listing the countries in alphabetical order.

Author, Journal (Year), Country	Study Design and Time of Study	Sample Size	Age Range or Mean +/- SD	Sex, Female No. (%)	Vaccination History	New thyroid disorders and complications No (%)	Comments
Razu MH, BMC Endocr Disord (2022), Bangladesh (14)	Cross sectional study Random selection 2021-2022	70	35.50 +/- 15.6	31 (44.9%)	30 vaccinated for COVID 19	30 (42.86%) non-thyroidal illness syndrome (unvaccinated COVID-19 test positive) 30 (42.86%) normal thyroid (vaccinated COVID-19 test negative) 10 (14.28%) normal thyroid (controls, healthy)	Thyroid function disruption happens between COVID-19 infection and vaccination phases Thyroid hormone levels change dynamically and recover gradually and spontaneously with COVID-19
					30 unvaccinated		
Burekovic A, Med Arch (2022), Bosnia and Herzegovina (15)	Retrospective-prospective study Jan 2019 - Dec 2021	58 (2019)	18-70 years	55 (94.8%)	Unknown	55 (94.83%) hypothyroidism 3 (5.17%) subclinical hypothyroidism	COVID-19 affects thyroid function leading to clinical and subclinical hypothyroidism, which may require hormone replacement
		89 (2020)	18-70 years	82 (92.1%)	Unknown	77 (86.52%) hypothyroidism 12 (13.48%) subclinical hypothyroidism	
		101 (2021)	18-70 years	93 (92.08%)	Unknown	93 (92.08%) hypothyroidism 8 (7.92%) subclinical hypothyroidism	
Yanachkova V, Biotechnology & Biotechnological Equipment (2023), Bulgaria (16)	Observational prospective study Jan 2021 - Jun 2021	113	43	78 (69%)	Unknown	2 months after COVID-19, 44 (38.9%) euthyroid 69 (61.1%) thyroid dysfunction: - 54 (78.3%) subclinical hypothyroidism - 6 (8.7%) overt hypothyroidism - 9 (13%) subclinical hyperthyroidism 3 months after COVID-19, 81 (71.7%) euthyroid 32 (28.3%) subclinical hyperthyroidism	COVID-19 affects thyroid function, triggers autoimmune thyroid disease and can lead to subclinical hypothyroidism
Barajas Galindo DE, Clin Endocrinol (2023), Castilla y León, Spain (17)	Cross sectional observational study 2017-2019 (Pre-pandemic) and 2020-2021 (Pandemic)	Pre-pandemic 81	48.11 +/- 16.51	158 (87.8%)	42/66 patients were vaccinated 90 days before symptom onset in the pandemic period	Graves’ Disease in each year. 27 (15.0%) in 2017 25 (13.9%) in 2018 29 (16.1%) in 2019 33 (18.3%) in 2020 66 (36.7%) in 2021	Increase in Graves’ Disease incidence in 2021, especially in women with a positive history of smoking COVID and vaccines can induce hyperactivation of immune system
		Pandemic 99					
		Total 180					
Chen M, Thyroid (2021), China (18)	Retrospective review Jan 2020 - Mar 2020	50	Not specified	Not specified	Unknown	28 (56%) lower than normal TSH (non-thyroidal illness syndrome)	Changes in serum TSH and T3 levels are an important manifestation of the courses of COVID-19 (more severe COVID equals lower TSH/T3 levels)
Wang W, Front Endocrinol (2021), China (19)	Retrospective study Jan 2020 - Mar 2020	84	57.3 +/- 14.5	31 (37%)	Unknown	52 (61.9%) thyroid function abnormalities	Thyroid function abnormalities are common in COVID-19 patients Thyroid function abnormalities are worse in severe COVID cases Recovery is gradual and spontaneous
Lui DTW, Endocr Pract (2021), China (20)	Prospective cohort study Jul 2020 – Dec 2020	204 all	55 (44.3-63.0)	109 (53.4%)	Unknown	43 (21.08%) Abnormal TFTs in acute COVID-19: - 35 (81.4%) recovered spontaneously - 13 (30.23%) subclinical thyrotoxicosis (10 of them spontaneously resolved) 21 (48.84%) isolated low fT3 levels (nonthyroidal illness): 19 recovered, 1 had painless thyroiditis and 1 was clinically ill 6 (13.95%) isolated mildly abnormal fT4 or fT3 levels; all subsequently normalized 3 (6.98%) patients with subclinical hypothyroidism (all had positive anti-TPO) 161 (78.92%) patients with normal TFTs in acute COVID-19, 3 (1.9%) had abnormal TFTs upon follow-up: - 1 subclinical hypothyroidism - 1 mildly elevated fT4 - 1 mildly elevated fT3	Thyroid dysfunction during acute COVID-19 usually resolved There is an increased incidence of anti-TPO positivity
		172 symptomatic in acute COVID-19 illness	56.0 (45.0-63.0)	90 (52.3%)			
		32 asymptomatic in acute COVID-19 illness	53.5 (32.0-64.5)	19 (59.4%)			
Lui DTW, J Clin Endocrinol Metab (2021), China (21)	Prospective cohort study Jul 2020 – Aug 2020	191	53.5 ± 17.2	92 (48.2%)	Unknown	25 (13.1%) abnormal TFTs 14 (7.3%) thyrotoxicosis 10 (5.2%) isolated low TSH (thyroiditis) 10 (5.2%) isolated low fT3 (nonthyroidal illness) 12 (6.3%) abnormal TSH	Higher CRP level was associated with low fT3 and a decrease in fT3 was correlated with deterioration of clinical condition or increasing severity of COVID-19
Lui DTW, Endocrine (2023), China (22)	Prospective follow up study COVID survivors Jul 2020 -May 2021	250	52.7 +/- 15.3	124 (49.6%)	None vaccinated	51 (20.4%) abnormal TFTs 1 (0.4%) Graves’ Disease & overt thyrotoxicosis 15 (6.0%) Subclinical thyrotoxicosis 1 (0.4%) Painless thyroiditis 25 (10.0%) hypothyroid with low T3 3 (1.2%) subclinical hypothyroidism 11 (4.5%) abnormal TFTs after interferon	Persistent thyroid function abnormalities in COVID-19 associated with abnormal TFTs COVID infection does not lead to change in autoimmunity, but interferon treatment is associated with a modest increase in antibody titers
Anaya JM, J Transl Autoimmun (2021), Columbia (23)	Convenience sampling, retrospective 2021	120 COVID-19	57.5	35 (29.2%)	Unknown	44 (36.7%) TPO Autoantibodies 2 (1.7%) TG Autoantibodies	Latent autoimmunity is common in patients with COVID-19 Anti-TPO antibodies were higher in COVID-19 patients compared to pre-pandemic controls
		100 healthy				20 (20%) TPO Autoantibodies 3 (3%) TG Autoantibodies	
Lui DTW, Endocrinol Metab (Seoul) (2021), Hong Kong (24)	Prospective study, July 21 to September 21, 2020	122	58 (44–63)	62 (50.8%)	Unknown	Abnormal TFTs on admission 20 (16.4%), and 15 (12.3%) recovered Baseline anti-TPO positivity 25 (20.5%) Baseline anti-Tg positivity 13 (10.7%) Increase in anti-thyroid peroxidase (TPO) (P<0.001) and anti-thyroglobulin (P<0.001), but not anti-thyroid stimulating hormone receptor titers (P=0.486)	Most patients with thyroid dysfunction on admission recovered during convalescence. An increase in anti-thyroid antibody titers post-COVID-19 warrants further follow-up
Lui DTW, Endocr Pract (2024), Hong Kong (25)	Retrospective, propensity-score matched, population-based study	84,034 COVID-19 survivors	61.0 (48.0-73.0)	49415 (58.8%)	68837 (81.9%) vaccinated	Thyroid dysfunction (HR 1.058, P = 0.154) Hyperthyroidism (HR 1.061, P = 0.345) Hypothyroidism (HR 1.062, P = 0.255) Initiation of antithyroid drug (HR 1.302, P = 0.070) Initiation of levothyroxine (HR 1.086, P = 0.426) Thyroiditis (HR 3.488, P = 0.252)	COVID-19 was unlikely to be associated with persistent thyroid dysfunction
		84,034 matched controls	62.0 (49.0-73.0)	50236 (59.8%)	70296 (83.7%) vaccinated		
Herczeg V, Eur J Pediatr (2023), Hungary (26)	Prospective, multicenter registry analysis, children Mar 2021- Mar 2022	452 out of 458	12.4 +/- 3.8	250 (54.6%)	52 after COVID	30 (6.6%) thyroid autoimmunity 8 (1.8%) isolated TSH elevation 18 (4.0%) ultrasound proven autoimmune thyroiditis	Higher rate of thyroid autoimmunity and autoimmune thyroiditis with previous COVID-19 infections Vaccination has no effect on the prevalence of thyroid autoimmunity
					18 before COVID		
					87 (19.2%) vaccinated 365 (80.8%) No vaccinated		
Arora S, Cureus (2022), India (27)	Single center retrospective study, Sept 2020 - Dec 2020	102	55.5 +/- 14.8	24 (23.5%)	Unknown	60 (58.8%) non-thyroidal illness 36 (35.3%) euthyroid state 5 (4.9%) thyrotoxicosis (2 patients died) 2 (1.9%) overt hypothyroidism	Low FT3 level is associated with severe disease and all-cause mortality New-onset thyrotoxicosis is secondary to subacute thyroiditis, but does not change the outcome
Mondal S, Postgrad Med J (2023), India (28)	Retrospective-prospective study, SAT within 3 months post COVID	11 SAT post COVID	44.09 +/- 16.6	7 (63.63%)	No vaccination history for COVID	670 patients with COVID-19 infection 160 patients with adequate follow-up data for 6 months 11 patients (6.8%) with COVID-19-associated thyroiditis 5 painless SAT and 6 painful SAT	Three months’ follow-up: 7 (63.7%) euthyroid, 3 (27.3%) subclinical hypothyroidism and 1 (9%) overt hypothyroidism Six months’ follow-up: 9 (81.8%) euthyroid, 1 (9%) subclinical hypothyroidism and 1 (9%) overt hypothyroidism
Lania A, Eur J Endocrinol (2020), Italy (29)	Single center retrospective study, Mar 2020 - Apr 2020	287	66 (27–92)	94 (32.8%)	Unknown	58 (20.2%) thyrotoxicosis (overt in 31 or 10.8%) 15 (5.2%) hypothyroidism (overt in 2 or 0.7%) 214 (74.6%) normal thyroid function	TSH values inversely correlated with age of COVID-19 patients and IL-6 Thyrotoxicosis significantly associated with higher IL-6
Brancatella A, J Endocr Soc (2021), Italy (32)	Cross-sectional, retrospective study, Jan 2016 - Dec 2020	198	44.6	167 (84%)	Unknown	Comparable SAT: 40 in 2016 34 in 2017 43 in 2018 35 in 2019 46 in 2020	There were no increased in total number of SAT in 2020 compared to the previous years
Pizzocaro A, Endocrine (2021), Italy (30)	Single center prospective study, Mar 2020 - Apr 2020	29	64 (43–85)	11 (37.9%)	Unknown	On admission: thyrotoxicosis 17 (58.62%) and subclinical thyrotoxicosis 12 (41.38%) Follow up (30–120 days): euthyroid 28 (96.6%) and hypothyroidism 1 (3.4%) Hypo-echogenicity on ultrasound of thyroid gland with higher TSH values 10 (34.5%)	Thyroid function spontaneously normalizes in most COVID-19 patients Ultrasound changes may predict thyroid dysfunction
Rossini A, Front Endocrinol (2023), Italy (31)	Single center prospective observational cohort study, May 2020 - Jul 2020	494 COVID-19 survivors, no thyroid autoimmunity	65 (55–73)	132 (26.7)	Unknown	85 (14.2%) TPO-Ab 43 (7.2%) Tg-Ab 23 (3.8%) both TPO-Ab and Tg-Ab 105 (17.5%) Thyroid autoimmunity 56/59 (94.9%) patients with positive antibodies had thyroiditis on US	Autoimmune thyroid disease prevalence in COVID-19 survivors doubled as compared to age & sex matched controls COVID-19 elicits thyroid autoimmunity, but a minority demonstrate TFT abnormalities
		105 COVID-19 survivors with thyroid autoimmunity	61 (54–72)	48 (45.7)			
		498 control	52.7	320 (64.2%)		Control group: 37/444 (8.3%) TPO-Ab 33/373 (8.8%) Tg-Ab 14/325 (4.3%) Both antibodies	
De Vincentis S, Eur Thyroid J (2024), Italy (44)	Prospective study, Nov 2020 - May 2022	58	50.0 (42.3–56.4)	47 (81%)	19 (32.8%), 1^st^ vaccine before SAT		No difference in therapeutic approach to SAT or outcome between COVID+ and COVID− groups
Adhikari P, JNMA J Nepal Med Assoc (2023), Nepal (33)	Cross sectional study Sept 2022 - Feb 2023	38	48	7 (30.43%)	Unknown	23 (60.5%) subclinical hypothyroidism 11 (28.9%) TSH > 10	COVID-19 may increase the risk of hypothyroidism and subclinical hypothyroidism
Malik J, PLoS One (2021), Pakistan (34)	Retrospective pilot study single center, Apr 2020 - Jul 2020, Total 76	48 COVID-19+	51 ± 19.30	17 (35.4%)	Unknown	36 (75%) abnormal thyroid functions in COVID-19 pneumonia	TSH and TT3 had significantly lower mean values in severe COVID-19 COVID-19 pneumonia changes TSH and T3 levels TT3 (P-value 0.01), IL-6 (P-value <0.01), and Procalcitonin (P-value 0.03) are independent risk factors for COVID-19
		28 COVID-19-	64.79 ± 11.44	13 (46.4%)		24 (85.7%) abnormal thyroid functions in non-COVID-19 pneumonia	
Elhadd T, Qatar Med J (2022), Qatar (35)	Case series report, single outpatient endocrine center Oct 2020 - July 2021	10	14-51	7 (70%)	Unknown	5 (50%) Graves hyperthyroidism. 2 (20%) Chronic hypothyroidism. 1 (10%) Subacute thyroiditis. 1 (10%) “Sick euthyroid disease,”. 1 (10%) Central hypothyroidism.	Female preponderance in most thyroid dysfunction after COVID-19 Complete remission in most patients Autoimmune thyroid triggering may require treatment
Ostapchuk VA, Romanian Journal of Diabetes Nutrition and Metabolic Diseases (2023), Romania (36)	Prospective cohort study, Mar 2020 - Sept 2020, Total 123 19 - 49 years	12	38.23 ± 4.61	123 (100%)	Unknown	12 subclinical hypothyroidisms + COVID-19	Patients with Autoimmune thyroiditis have experienced structural changes in the thyroid gland and reduced thyroid hormone synthesis after COVID-19 infection
		31	37.71 ± 4.07			31 subclinical hypothyroidisms, no COVID-19	
		32	39.27 ± 3.12			32 hypothyroidisms + COVID-19	
		48	36.18 ± 2.73			48 hypothyroidisms, no COVID-19	
Mukhtar N, Endocr Metab Sci (2022), Saudi Arabia (37)	Prospective follow up study, May 1-20, 2020	50	47 (25–58)	21 (42%)	Unknown	5 (10%) non-thyroidal illness syndrome 45 (90%) euthyroid	Thyroid dysfunction in COVID is rare, mild, and transient
Ahn HY, Thyroid (2022), South Korea (38)	Retrospective, cross-sectional population-based study	15,447 3607 in 2017 3582 in 2018 3995 in 2019 4263 in 2020	10-80	12,963 (83.9%)	Unknown	Incidence rates per 100,000 persons for women/men: 2017, 11.9/2.2 2018, 11.6/2.3 2019, 12.9/2.6 2020, 14.0/2.6	Subacute thyroiditis incidence was significantly higher in 2020 than in 2017–2019 The increased SAT incidence in 2020 is probably associated with SARS-CoV-2 infection, because SAT-related viral infections decreased in 2020
Batman A, J Clin Endocrinol Metab (2023), Turkey (39)	Nationwide, multicenter, retrospective cohort (53 endocrinology centers in Turkey), March 2020-April 2022 Total 811	Cont-SAT 455	42 (37–49)	338 (74.3%)		455 (56.1%) in the Cont-SAT group (Classic subacute thyroiditis)	Clinical characteristics, hypothyroidism or recurrence outcomes were not significantly different between subacute thyroiditis etiology groups.
		Cov-SAT 98	41 (36–50)	64 (65.3%)		98 (12.1%) in the Cov-SAT group (COVID-19–related subacute thyroiditis)	
		Vac-SAT 258	42 (36–49)	187 (72.5%)		258 (31.8%) in the Vac-SAT group (SARS-CoV-2 vaccine–related subacute thyroiditis)	
Khoo B, J Clin Endocrinol Metab (2021), UK (40)	Cohort observational study Mar 2020 - Apr 2020 Total 456	334 COVID-19 positive	66.1	131 (39.2%)	Unknown	289 (86.5%) Euthyroid No hyperthyroidism 2 (0.6%) Hypothyroid 18 (5.4%) Subclinical hyperthyroidism 17 (5.1%) Subclinical hypothyroidism 8 (2.4%) Secondary hypothyroidism	Most patients with COVID-19 present with euthyroidism Nonthyroidal illness syndrome (mild reductions in TSH and FT4) in COVID-19 patients was observed Return to baseline observed in COVID-19 survivors
		122 COVID-19 negative	63.8	54 (44.3%)		106 (86.9%) Euthyroid No hyperthyroidism or hyperthyroidism 8 (6.6%) Subclinical hyperthyroidism 7 (5.7%) Subclinical hypothyroidism 1 (0.8%) Secondary hypothyroidism	
Clarke SA, J Clin Endocrinol Metab (2021), UK (41)	A prospective, observational study March to November 2020	70	55.9	23 (32.9%)	Unknown	Normal TFT ≥ 3 months after COVID-19 in patients without preexisting thyroid disease (68)	Thyroid function ≥ 3 months after presentation with COVID-19 was normal
McCowan R, Front Endocrinol (2022), UK (42)	Retrospective study Jan 2016 - Dec 2021 Total 244	Pre-pandemic 174	11.5 (5 - 16.9)	114 (65.5%)	Unknown	33 (77%) Hyperthyroid (Pre-pandemic) 141 (70%) Hypothyroid (Pre-pandemic)	Increase in rates of transient thyroid dysfunction during COVID-19 pandemic
		Pandemic 70		49 (70%)		10 (23%) Hyperthyroid (Pandemic) 60 (30%) Hypothyroid (Pandemic)	
Donner JR, Endocr Pract (2023), USA (43)	Retrospective chart review (0–18) Jan 2018 - Feb 2020 (Pre-pandemic)	18	13.8	14 (77.8%)		18 Graves’ Disease (Pre-pandemic) 1.2% of all new endocrine visits pre-pandemic	Increased incidence of new onset pediatric Graves’ disease (GD) during the first 2 years of COVID-19 Increased severity of GD during the pandemic
	Mar 2020 - Dec 2021 (Pandemic)	33	13.9	27 (81.8%)	7 (21.2%) COVID received vaccine prior to GD onset	33 Graves’ Disease (Pandemic) 2.6% of all new endocrine visits during pandemic	
Bogojevic M, Clin Endocrinol (2024), USA (45)	Observational cohort	20,366	70 (59.0,80.0)	1048 (65%)	Unknown	Pre-existing hypothyroidism was not associated with severe disease, ICU admission or ICU mortality	Hypothyroidism 1616 cases (7.9%)
			62 (49.0,73.0)	7809 (42%)	Unknown		No hypothyroidism 18,750 cases (92.1%)

Quality assessment was performed using ROBINS (Risk of Bias in Non-randomized Studies of Exposure) or the involvement of a third author. All studies are nonrandomized studies, which may introduce a considerable risk of bias into the review. We generated the review questions, produced review-specific guidance, constructed a flow diagram for the study selection, and then judged bias and applicability. We looked at the following domains: sample size, age, sex, vaccination history, follow-up duration, study design, methods of case selection, outcome measurements, and interventions. We found significant heterogeneity among the studies after considering a subgroup evaluation for before mentioned variables, especially sample size, age, sex, vaccination history, follow-up duration, and study design. Uncovered biases remain, including accessibility to healthcare, medication availability, and therapeutic approaches to viral treatment, which technically can affect the outcomes of the studies.

### Strategy for data interpretation

We prepared a table to show the results of selected studies, and then discussed the differences in the results of studies without running statistical analysis on detailed data. Meta-analysis was not performed because of the heterogeneity among selected articles and the lack of a tool to overcome the effects of uncovered biases on outcomes. There was no limit to manuscript selection.

## Results

A total of thirty-two studies were carefully chosen during the selection process (14–45). The eligible manuscripts include 13 prospective and 19 retrospective or cross-sectional studies. An observational study by Fallahi et al. from Italy during the first phase of the pandemic in 2020, reported that patients with autoimmune thyroid disease have higher prevalence of SARS-CoV-2 infection (46). The prevalence of thyroid dysfunction was reported as 15% in a systematic review that evaluated 30 cohort studies and included 9,707 COVID-19 cases. Noticeably, thyroid dysfunction prevalence correlated with severity of COVID-19 (6.2% among mild to moderate cases and 20.8% among sever cases) (47). Another meta-analysis by Ashrafi in January 2024, reported 26% prevalence of non-thyroidal illness syndrome (NTIS) and 10% prevalence of thyrotoxicosis. The aforementioned study selected 8 out of 1,256 studies and included 1,654 participants. The prevalence of hypothyroidism (3%), isolated elevated FT4 (2%), and isolated low FT4 (1%) were unremarkable (48). NTIS is the most common abnormality seen in the literature following acute SARS-CoV-2 infection. Typical findings in NTIS include reduced triiodothyronine (T3) with normal or decreased thyroid stimulating hormone (TSH). The prevalence of NTIS in patients with COVID-19 ranges from 5 to 58 percent in the literature (21, 27). NTIS is also referred to as isolated low T3 syndrome and sick euthyroid syndrome. NTIS is reported from various areas, including Bangladesh (14), China (18–22), Hong Kong (24, 25), India (27, 28), Qatar (35), Saudi Arabia (37), and the United Kingdom (40–42).

Thyroiditis is another complication of SARS-CoV-2 infection, and includes SAT, painless thyroiditis, autoimmune thyroiditis and atypical thyroiditis (21, 49, 50). There is generally an increased risk of thyroiditis, thyroid autoimmunity, and autoimmune thyroiditis following acute SARS-CoV-2 infection, which is unlikely associated with persistent thyroid dysfunction, but may result in new-onset thyrotoxicosis or hypothyroidism (25–27, 31, 35, 36, 39, 51, 52). The reported incidence of thyroiditis and outcomes following acute SARS-CoV-2 infection varies by country, which could be due to the differences in population characteristics, severity of infection, and therapeutic interventions.

SAT typically present with fever, neck pain and symptoms and signs of thyrotoxicosis. The clinical manifestations of SAT following SARS-CoV-2 infection appear similar to the typical SAT (53). If acute SARS-CoV-2 infection is considered as a risk factor for SAT, then the incidence of SAT would be expected to rise after the pandemic. A nationwide study from South Korea found an increased incidence of SAT during the early phase of COVID-19 in 2020 (38). However, retrospective single-center studies reported that the incidence of SAT was not increased in Italy (32, 54) and Turkey (55, 56) during the COVID-19 pandemic. In addition, a multicenter nationwide study reported no differences in clinical characteristics or outcomes of SAT in Turkey (39) and no significant increase in thyroiditis in Hong Kong (25) after the pandemic. Finally, it seems that SARS-CoV-2 infection does not affect the onset, progression, and outcome of SAT as demonstrated by a multicenter prospective study from Italy that included 52 patients (7 COVID+ and 45 COVID-) (44).

Painless thyroiditis and autoimmune thyroiditis have also been reported following SARS-CoV-2 infection. In Hungary, rates of autoimmune thyroiditis were higher in the pediatric population with a history of SARS-CoV-2 infection (26). Data from Italy described an increased, almost doubled, rate of autoimmune thyroid disease prevalence in COVID-19 survivors, but a minority demonstrate thyroid function test abnormalities (31). SARS-CoV-2 infection can generally trigger autoimmunity as reported from Bulgaria (16), Columbia (23), Italy (31), Qatar (35), and Romania (36).

Atypical thyroiditis was reported by Muller et al. in 2020. It was reported in ICU patients with COVID-19 who presented with low TSH and T3 accompanied by normal or elevated thyroxine (T4) (50). This presentation is a combination of NTIS and thyrotoxicosis, which has been reported as T4 thyrotoxicosis previously (57).

Graves’ disease and Hashimoto’s thyroiditis are other possible complications of SARS-CoV-2 infection. There is a link between acute SARS-CoV-2 infection and Graves’ disease (17, 29). The first two cases of Graves’ disease after COVID-19 were reported in October 2020 (58). The epidemiologic studies about the prevalence of Graves’ disease during the COVID-19 pandemic are limited. An observational study from Spain described a significant increase in the incidence of Graves’ disease during the pandemic (17). In the United States reported an increased incidence of new-onset pediatric Graves’ disease during the first 2 years of COVID-19 (43). A retrospective cohort study from Taiwan that included 1,379,311 COVID-19 patients and 6,896,814 non-COVID-19 patients, reported an increased risk of thyroid dysfunction, including thyrotoxicosis and hypothyroidism, secondary to COVID-19. The risk of thyroid dysfunction, both thyrotoxicosis and hypothyroidism, following COVID-19 infection appears to be higher in older patients (aged 65 and above) and female (52). There is also a connection between SARS-CoV-2 infection and complications of Graves’ disease, such as orbitopathy (59, 60), thyrotoxic periodic paralysis (61–64) and thyroid storm (65–67). In terms of hypothyroidism, a bi-directional Mendelian randomization study supports a causal relationship between the host response to SARS-CoV-2 infection and increased risk of hypothyroidism (51). There are also studies from Bosnia and Herzegovina (15), Bulgaria (16), Hong Kong (25), India (27), Italy (30), Nepal (33), and Qatar (35) that reported development or increased risk of hypothyroidism after SARS-CoV-2 infection.

There are multiple studies investigating the correlation between pre-existing autoimmune thyroid diseases and the outcomes of COVID-19 (45, 68–73). Underlying thyroid disorders, especially hypothyroidism, are reported to be associated with a worse prognosis of COVID-19 in two systemic reviews from Indonesia in 2021 (74) and 2022 (75). Moreover, a large retrospective cohort from Turkey (total n=14,966; hypothyroidism n=8813; hyperthyroidism n=1822; normal thyroid function n=4331) showed a positive correlation between pre-existing hyperthyroidism or hypothyroidism and COVID-19 mortality (72). However, a large observational cohort (20,366 adult patients; pre-existing hypothyroidism in 1,616) from the USA showed that pre-existing hypothyroidism in hospitalized COVID-19 patients was not associated with worse outcomes of acute SARS-CoV-2 infection (45). In addition, two large population-based cohort studies are reassuring and suggested that hypothyroidism or hyperthyroidism were not associated with an increased risk of infection or worse outcomes in COVID-19 patients (69, 71).

Finally, it is important to mention that thyroid function testing has relatively good prognostic value for illness severity and predicting mortality risk in hospitalized moderate-to-severe COVID-19 patients (76).

## Discussion

Although the mortality and morbidity seen during the height of the pandemic has diminished, the SARS-CoV-2 infection has both short and long-term complications. Further efforts to improve the outcomes of acute SARS-CoV-2 infection are necessary. Acute SARS-CoV-2 infection causes transient and permanent thyroid dysfunction. Transient thyroid dysfunction is associated with COVID-19 severity, hospitalization and even mortality (77, 78). Permanent thyroid dysfunction leads to long-term medication use and worsening of underlying medical comorbidities. COVID-19 also increases the risk of autoimmunity against the thyroid gland, which may lead to thyroiditis, hypothyroidism or hyperthyroidism (52).

**Figure 2:**
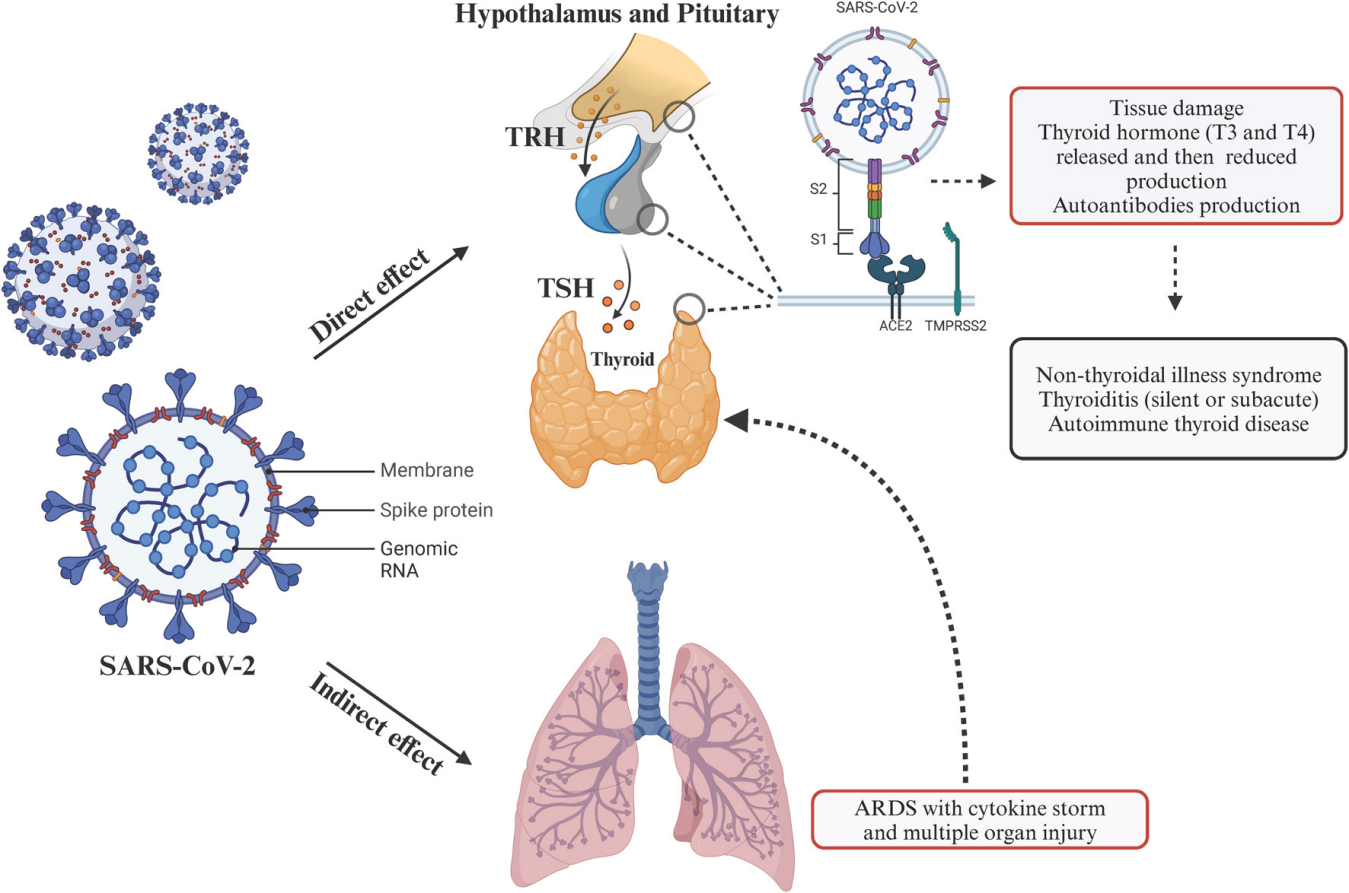
The diagram demonstrates the cascade of events initiated by SARS-CoV-2 infection leading to thyroid dysfunction. ACE2 and TMPRSS2 are expressed at the level of the hypothalamic-pituitary-thyroid (HPT) axis, facilitating SARS-CoV-2 entry into thyroid tissue and the HPT axis, and subsequent tissue injury. Additionally, SARS-CoV-2 infects lungs triggering cytokine storm, which is associated with organ dysfunction including thyroid and HPT axis. Created with BioRender.com.

The pathophysiologic and molecular mechanisms of thyroid dysfunction following acute SARS-CoV-2 infection have not been fully elucidated. SARS-CoV-2 enters into cells after attaching to cell receptor, predominantly ACE2. Subsequently, viral particle approximation, fusion and internalization are essential for viral RNA genome engulfment and viral replication in host cells. The processes of approximation and fusion are mediated by host proteases, such as TMPRSS2 and ADAM17 (11). There is evidence regarding the ability of SARS-CoV-2 to directly assault the thyroid gland including the presence of ACE2 and TMPRSS2 in the thyroid tissue (79), along with detection of SARS-CoV-2 infection in follicular thyroid cells (80) and upregulation of immune genes is the SARS-CoV-2-positive thyroid specimens (81). This evidence not only suggests the ability of SARS-CoV-2 to directly invade thyroid tissue, but also proposes another mechanism of tissue injury by heightening inflammation through pro-inflammatory cytokines (80–82). Therefore, SARS-CoV-2 may disrupt thyroid function by entering thyroid cells, replicating locally, and triggering autoimmunity (83). ACE2 and TMPRSS2 expression were also found in the hypothalamus and pituitary, which makes them susceptible to direct damage following acute SARS-CoV-2 infection (84). The presence of ACE2 on hypothalamus, pituitary and thyroid tissue could be associated with not only triggering autoimmunity against thyroid by SARS-CoV-2, but also affecting the hypothalamic-pituitary-thyroid (HPT) axis. Acute SARS-CoV-2 infection can also cause a hyper-inflammatory immune response or cytokine storm following pneumonia. The uncontrolled inflammatory response can damage host cells, which could potentially affect the function of thyroid gland or HPT axis too (82, 85). Moreover, it has been reported that pre-existing thyroid conditions may worsen COVID-19 outcomes. Therefore, a bidirectional relationship between acute SARS-CoV-2 infection and thyroid dysfunction may exist (**Figure 2**).

Furthermore, we must underscore the challenges in diagnosing thyroid complications after COVID-19, which are sometimes associated with overlapping symptoms and laboratory findings. Additionally, the increased risk of autoimmunity may precipitate development of long COVID syndrome (7). Long COVID is a multisystem condition that develops in almost 10% of infected patients, especially following a severe acute SARS-CoV-2 infection. Thyroid dysfunction appears unlikely, but has a possible correlation with long COVID. The heterogeneity of symptoms, and severity and duration of long COVID is sometimes associated with missed diagnosis and delayed treatment of potential preventable conditions such as thyroid dysfunction (7, 85). However, the data about correlation between thyroid dysfunction and long COVID are conflicting. It has been reported that there is a correlation between thyroid or pituitary dysfunction and long COVID (25, 86). However, multiple cohort studies reported that there is no meaningful correlation between long COVID and thyroid dysfunction (20, 22, 25, 41).

The current review has several limitations. Our review was limited to the studies written in the English language. The review excludes more recent publications completed after our initial review (March 31, 2024). We also reviewed information from different parts of the world, but there were still some missing areas, which limited the global applicability of the results. In addition, the differences in studies’ results may be caused by the evolution of treatment modalities for COVID-19 throughout the pandemic, the time of data collection in different studies and momentary incidence of the COVID-19 related complications.

The current recommendations for treating acute SARS-CoV-2 infection is primarily antivirals, such as remdesivir, molnupiravir and nirmatrelvir-ritonavir. Antivirals are designed to improve symptoms, reduce the duration of disease and prevent complications, such as hospitalization and post-acute sequelae of SARS-CoV-2 infection. However, SARS-CoV-2 continuously changes by altering the genetic codes, and routine usage of antivirals can induce new mutations too. For this reason, SARS-CoV-2 evolves gradually by accumulating mutations that can cause resistance to current antivirals and quicker spread of new variants with increased virulence (11, 87). Moreover, there are examples of hypothetical theories about the pathophysiology of post-acute sequelae of COVID-19, including sustained viral replication, presence of viral particles in organs, permanent inflammatory response, endothelial dysfunction, and altered immune function with a tendency toward autoimmunity (88, 89). Applying an alternative approach by targeting receptors, reducing virus engulfment and modulating immune response would be reasonable to improve COVID-19 outcomes.

Current literature supports the usefulness of dipeptidyl peptidase 4 inhibitors (DPP-4 inhibitors), metformin, spironolactone, and ursodeoxycholic acid (UDCA) in reducing virus entry into the cells and alleviating inflammatory responses (1). Each of the above medications has potential benefits to improve the clinical outcome of a patient with acute SARS-CoV-2 infection, and are not impacted by viral mutations. DPP-4 inhibitors are not only immunomodulators but also reduce SARS-CoV-2 interaction with receptors and diminish viral replication (11, 90). Spironolactone has anti-inflammatory and anti-thrombotic effects, and plays protective roles against SARS-CoV-2-mediated endothelial dysfunction by preventing damage to endothelial glycocalyx (91). Metformin reduces viral load, inflammation and thrombotic risks (92). Combining these medications can provide different valuable defenses against SARS-CoV-2 simultaneously, enhance the efficacy of treatment and further reduce complications of acute SARS-CoV-2 infection. In support of this statement, it has been shown that the combination of spironolactone and sitagliptin could reduce hospitalization of acute SARS-CoV-2 infection by almost 78 percent, which was superior to antivirals by some means (11, 93).

In conclusion, COVID-19 is associated with disruption of thyroid function, such as NTIS and thyroiditis. The preferred treatment options for COVID-19 may need to change as studies identify more promising medications that target the SARS-CoV-2 receptor. The use of antivirals seems inadvisable to prevent complications of acute SARS-CoV-2 infection. DPP-4 inhibitors, metformin, and spironolactone are relatively safe medications, which may be added to antivirals or used in combination for acute SARS-CoV-2 infection based on clinical judgment. It is crucial to evaluate these medications in clinical trials and produce more evidence to support their future use.
